# When Size Matters: New Insights on How Seed Size Can Contribute to the Early Stages of Plant Development

**DOI:** 10.3390/plants13131793

**Published:** 2024-06-28

**Authors:** Alessandra Boccaccini, Sara Cimini, Hira Kazmi, Andrea Lepri, Chiara Longo, Riccardo Lorrai, Paola Vittorioso

**Affiliations:** 1 Department of Science and Technology for Sustainable Development and One Health, Università Campus Bio-Medico di Roma, via Álvaro del Portillo, 21, 00128 Rome, Italy; a.boccaccini@unicampus.it (A.B.); s.cimini@unicampus.it (S.C.); 2 Department of Biology and Biotechnology “Charles Darwin”, Sapienza University of Rome, 00185 Rome, Italy; hira.kazmi@uniroma1.it (H.K.); andrea.lepri@uniroma1.it (A.L.); chiara.longo@uniroma1.it (C.L.); riccardo.lorrai@uniroma1.it (R.L.)

**Keywords:** seed size, germination, cell wall, environment, development, *Arabidopsis thaliana*, *Oryza sativa*

## Abstract

The seed habit is the most complex and successful method of sexual reproduction in vascular plants. It represents a remarkable moment in the evolution of plants that afterward spread on land. In particular, seed size had a pivotal role in evolutionary success and agronomic traits, especially in the field of crop domestication. Given that crop seeds constitute one of the primary products for consumption, it follows that seed size represents a fundamental determinant of crop yield. This adaptative feature is strictly controlled by genetic traits from both maternal and zygotic tissues, although seed development and growth are also affected by environmental cues. Despite being a highly exploited topic for both basic and applied research, there are still many issues to be elucidated for developmental biology as well as for agronomic science. This review addresses a number of open questions related to cues that influence seed growth and size and how they influence seed germination. Moreover, new insights on the genetic–molecular control of this adaptive trait are presented.

## 1. Introduction

The remarkable significance of seeds was first emphasized by Darwin, who used seeds as an example to explain three concepts of the theory he was developing: competition, adaptation, and natural selection [1].

The evolution of seeds in the late Devonian gave rise to a widespread diffusion of plants on earth, as reproduction became water-independent due to the development of the pollination process. The following establishment of angiosperms, with respect to the gymnosperms, was due to the double fertilization-based reproduction mechanism, which gave rise to three distinct tissues in the seed: the maternal seed coat (testa), the triploid endosperm, and the diploid embryo [2,3,4,5]. The latter gives rise to seedlings and will be fed with nutrients by the endosperm until germination. As for the seed coat, which arises from the sporophytic integuments, as well as protecting the embryo, it plays a key role in determining the seed size [6].

Seed size is a complex quantitative and multigenic trait that is strictly regulated; given the diverse origin of seed tissues, there is both a maternal and zygotic control of this character, as well as a crosstalk between these molecular networks, allowing fine-tuning control of this developmental process [7,8,9]. Although this trait is genetically determined and, as such, conserved within a species [10], it is influenced by environmental conditions, such as temperature, day length, and light.

The maternal integuments delimit the cavity where both the embryo and the endosperm will develop, thus playing a key role in defining the final dimension of the seed [11,12,13]. The maternal control of seed size relies on several mechanisms, such as the ubiquitin-proteasome and the G-protein pathways [13,14,15,16,17,18], as well as the mitogen-activated protein kinase (MAPK) signaling [19], phytohormones, and growth factors [20].

In rice, as in Arabidopsis, several major QUANTITATIVE TRAIT LOCI (QTLs) associated with seed size variation have been mapped. Very recently, one of these QTLs for seed size and weight has been identified on chromosome 1 (SSW1, SEED SIZE, and WEIGHT1) [21]. SSW1 encodes an amino acid transporter, AAP8, which has been previously shown to be involved in the source-to-sink partitioning of organic nitrogen [22] and in seed development [23]. SSW1 positively affects seed size, as it alters cell proliferation of the maternal integuments of the ovules and developing seeds [21]. Natural variation in SSW1 results in the dominant *SSW1^Cvi^* allele characterized by larger seeds and increased seed yield with respect to the recessive *SSW1^Ler^* allele. In addition, the amount of amino acids and storage proteins were higher in the line expressing the *SSW1^Cvi^* allele compared to the one bearing the *SSW1^Ler^* allele, consistent with the amino acid permease activity of SSW1. Given the increased amino acid transport activity of the *SSW1^Cvi^* allele, the nitrogen use efficiency (NUE) also increased under low nitrogen conditions [21], thus defining a link between an environmental situation and an endogenous molecular mechanism.

Among the hormones involved in the control of seed size and yield, gibberellins (GA) have been proposed both as a growth-promoting hormone and for the effects of overexpression of some *GA-stimulated Arabidopsis* (*GASA*) genes, namely *GASA4* and *GASA10*, which caused an increase in seed size and weight [24,25]. Furthermore, the DELLA gain-of-function mutants *gai1* and *rgl2Δ17*, which are insensitive to GA [26,27], have been shown to affect seed size and morphology [28,29]. Very recently, it has been demonstrated that DELLA proteins contribute to increased cell numbers in maternal integuments through the induction of the transcription factor AINTEGUMENTA, which promotes cell proliferation in these tissues [30,31].

Organ size is an important trait of plant ecology, and in crops, it is directly related to yield and quality. In particular, presently, seed size plays an important role due to constantly changing climate conditions. In fact, despite breeding efforts, many phases of the crop life cycle remain sensitive to adverse environmental cues, with the final effects of decreasing the size, number, and fertility of seeds, delaying germination, and reducing seed vigor in crops [32]. Therefore, gaining insights into the determinants of this multigenic trait is of concern for both basic and applied research.

The genetic and molecular basis of the control of seed size has been recently reviewed [33] both in monocots and dicots; therefore, in this review, we will address a number of open questions directly or indirectly related to the genetic and molecular control of seed size.

**Figure 1:**
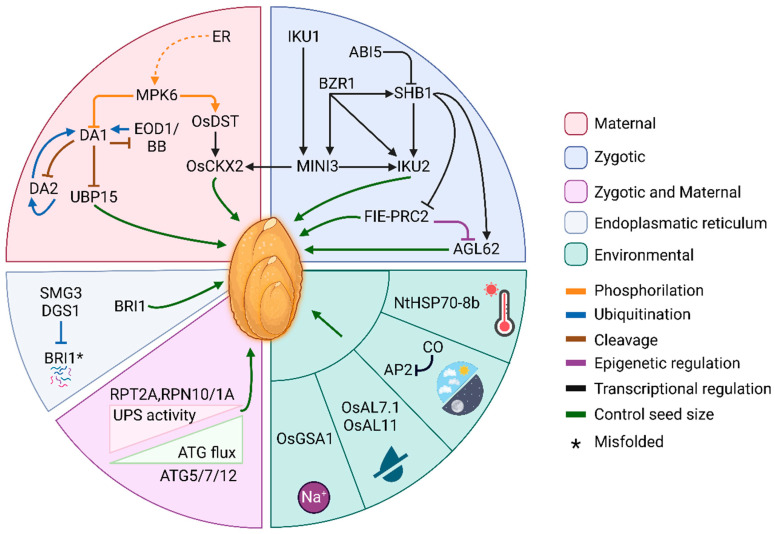
Mechanisms and pathways to control seed size. The figure shows the different regulators described in the review that act through maternal and/or zygotic tissues and in response to environmental stresses. All regulators have been studied in Arabidopsis unless otherwise specified. The dashed lines represent indirect interaction. Created with BioRender.com (accessed on 6 June 2024).

In particular, following an analysis of very recent insights into the molecular mechanisms that control this multigenic character, we will discuss the role of the cell wall as a constraint on seed growth, but also the effect of environmental conditions on seed development and final size. Then, agricultural implications will be considered, as seed size influences seed yield, thus representing a basic adaptive trait for crops. Finally, we will also focus on the interplay between seed size and seed germination, examining if and how the former influences the germination process and affects germination rate and/or timing (Figure 1).

## 2. Developmental Control of Seed Size

### 2.1. Maternal and Zygotic Control of Seed Size

Maternal and zygotic tissues cooperate in controlling the growth of the embryo, the endosperm, and the seed coat, thus defining seed size. Given that two recent reviews summarize the genetic and molecular networks underlying the control of seed size [33,34], in this section, some new insights on the best characterized maternal and zygotic regulation pathways, namely the ubiquitin-proteosome and the HAIKU ones, will be discussed (Figure 1).

The ubiquitin-proteasome pathway targets key factors regulating the growth and development of maternal tissues involved in seed formation [9,34]. In Arabidopsis, this pathway influences seed size through the activity of the ubiquitin receptor DA1 [14] and of its closest homolog DA1-related (DAR1). DA1 is a ubiquitin receptor with two Ubiquitin-Interacting Motif (UIM) domains and a C-terminal peptidase activity. The *da1-1* allele produces a mutant protein DA1^R358K^, which negatively affects DA1 and DAR1 and produces larger seeds. The effects of R358K mutation in an invariant amino acid in a conserved region could be explained by interfering with the interaction of DA1 family members with a common target [14]. Furthermore, two RING-type E3 ligases, DA2 and ENHANCER OF DA1 (EOD1)/BIG BROTHER (BB), physically interact with DA1 cooperating on its negative regulation of seed size. Consistently, *da2-1* and *eod1/bb* mutants have larger seeds and organs and synergistically enhance the *da1-1* phenotype [13,14,17,35]. Both DA2 and BB/EOD1 are able to monoubiquitinate DA1 [17], and, on the other hand, DA1 is able to cleave BB/EOD1 and DA2, which is likely a feedback regulation of the E3 ubiquitin ligases. Very recently, it has been proved that DA2 is able to auto-ubiquitinylate, and this affects its enzymatic activity towards DA1. Since the growth-inhibiting function of DA2 is dependent on DA1, this suggests a fine-tuning mechanism of the balance between cell proliferation and differentiation to regulate plant organ size [36]. The active DA1 cleaves downstream growth regulators as the Ubiquitin-specific Protease Target (UBP15), known as a growth-promoting factor in seeds. *ubp15* mutant was isolated as a suppressor of the *da1-1* mutant phenotype, while *UBP15* overexpressing plants mimic the seed size phenotype of *da1-1* [17].

Recently, new insights on this molecular pathway have been provided. Indeed, it has been established that the membrane receptor-like kinase ERECTA (ER) transmits to DA1 and other downstream effectors the signal about the regulation of seed size, revealing a nearly complete ER-MKK4/5-MPK3/6-DA1-UBP15 signaling pathway promoting the proliferation of outer integuments in developing seeds [37]. Since the *Landsberg erecta* (Ler) ecotype exhibits smaller seeds than the Columbia (Col-0), the authors investigated whether ER might be responsible for this difference. Evidence from a previous study in rice supports this hypothesis, as inactivation of the rice ER homolog (*OsER1*) resulted in a higher number and reduced size of grains in the spikelet [38]. Consistently, Ler plants expressing *gER^Col^* exhibited increased seed size, and *er105* mutant (Col-0) expressing *ERpro:ER-4myc* transgene reverted their smaller seeds to the WT size. ER controls seed size maternally by regulating cell proliferation, as it does for the growth of other organs [39,40]. Moreover, genetic and molecular data proved that the ER signal is carried by the DA1-UBP15 pathway. Indeed, *da1-1 mutation* and *UBP15-OX* restored the reduced size and outer integument cell number of the *er105* and *er105 erl2-1* mutant seeds, as the *ubp15-1* mutation restored the phenotype of the *ER-OX* seeds [37].

As for many plant growth processes [41,42,43], MKK4/5-MPK3/6 signaling acts downstream ER also for seed size control by phosphorylation and inactivation of DA1. MPK3 and MPK6 directly interact with DA1 in yeast two-hybrid assays, and MPK6 phosphorylates and inactivates DA1. Indeed, both WT and non-phosphorylatable DA1 proteins recovered the phenotype of *da1-1* seeds, while the constitutively phosphorylated DA1 only slightly decreased the size and outer integument cell number of *da1-1* seeds. Interestingly, in the signaling pathway that regulates seed size, ER does not need its cytoplasmic domains, indicating that ER might function as a co-receptor. Indeed, the expression of gER^Col^ with a truncated cytoplasmic tail in *er105* mutant plants resulted in seeds similar to or larger than the WT [37].

The DA1 pathway is conserved among different species. For example, the overexpression of the negative interfering mutant of *Arabidopsis DA1* (*AtDA1^R358K^*) increased seed size and yield in *Brassica napus* [44]. Similarly, in maize, the overexpression of mutated *ZmDA1* or mutated *ZmDAR1* increases kernel yield [45]. 

The rice homolog of *AtER*, *OsER1*, has been linked to grain number and size. It has been demonstrated that OsER1 acts upstream of the OsMKKK10-OsMKK4-OsMPK6 cascade to control the number of spikelets per panicle and that this pathway regulates cytokinin metabolism. Indeed, OsMPK6 interacts with and phosphorylates the zinc finger transcription factor DROUGHT AND SALT TOLERANCE (DST), enhancing its transcriptional activity and, in turn, inducing expression of *CYTOKININ OXIDASE 2 (OsCKX2)*, which results in cytokinin degradation, thus maintaining cytokinin at the level required for spikelet formation [38]. Furthermore, the *Oser1* loss-of-function mutant produced shorter but wider grains. Nonetheless, the molecular mechanism responsible for grain shape controlled by OsER1 remains unclear, possibly due to the diverse downstream substrates of OsMPK6. Interestingly, OsDA1, a rice homolog of AtDA1, is a negative regulator of rice grain size and directly interacts with OsUBP15, which, similarly to Arabidopsis, positively regulates grain size [46]. It would be interesting to investigate whether the DA1-UBP15 pathway functions in a similar way in Arabidopsis and rice. Moreover, despite the importance of the DA1-UBP15 pathway in regulating seed size, the upstream elements of this pathway are not yet known.

Endoplasmic reticulum (ER)-associated degradation (ERAD) ubiquitin-proteasome system is required for the degradation of misfolded proteins in the ER [47]; therefore, its E1 ubiquitin-activating enzymes are ER-associated [48]. Recently, it has been shown that *SMALL GRAIN 3* (*SMG3*), which encodes an ERAD-related E2, is involved in grain size and weight in rice (Li et al., 2023). Furthermore, DECREASED GRAIN SIZE 1 (DGS1), previously linked to grain size regulation (Zhu et al., 2021), is an active E3 ubiquitin ligase that interacts with SMG3 in ER. Indeed, overexpression of both *SMG3* and *DGS1* resulted in longer grains [49]. Interestingly, both the *smg3* and *dgs1* mutants were less sensitive to brassinosteroid (BR), suggesting that SMG3 and DGS1 are involved in BR signaling. Moreover, DGS1 directly interacts with and ubiquitinates BRASSINOSTEROID INSENSITIVE 1 (BRI1), affecting its accumulation. Consistently, genetic analysis demonstrated that SMG3, DGS1, and BRI1 cooperate in regulating grain size and weight. The proposed working model explained how SMG3 and DGS1 influence grain size, at least in part, by mediating the degradation of misfolded BRI1, which may disturb the formation and/or function of normal BRI1 protein [49].

The ubiquitin (Ub)–26S proteasome system (UPS) consists of the core protease 20S and the 19S regulatory particle (RP). The *Arabidopsis* RPT2, an RP subunit, is encoded by two homologous genes, *AtRPT2a* and *AtRPT2b*. A loss of function of *AtRPT2a* affected UPS activity, resulting in increased seed size and organs, such as leaves, stems, flowers, and embryos. These size abnormalities have been explained by the increased cell expansion, which compensates for a reduction in cell number [50]. More recently, UPS and autophagy interplay have been shown as essential for proper embryo and seed development, thus through maternal and zygotic tissues, by playing a crucial role in regulating proteome stability [51]. Three proteasome and four autophagy mutants have been analyzed to investigate their role in seed development. These mutants showed different seed size phenotypes: the proteosome (*rpn10-1*) and autophagy mutants (*atg10-1* and *atg13a-2 atg13b-2*) showed enlarged seed phenotype similar to *rpt2a-2*, while other UPS (*rpn1a-4*) and autophagy (*atg5-1* and *atg7-2*) mutants showed smaller, similar or larger variation in seed sizes compared to wildtype, respectively [51].

Moreover, the expression of UPS subunits with the total protein ubiquitylation has been compared, and the activity of critical enzymes involved in autophagy flux has been analyzed in developing seedlings, siliques, and embryos of *Arabidopsis thaliana.* The UPS activity decreased rapidly during early silique and embryo development, while autophagy flux was more sustained and seemed to play a role in later stages of seed development. Furthermore, the reduction of proteasome subunits during siliques development was less pronounced in autophagy-deficient mutants (*atg7-2*) or wildtype treated with autophagy inhibitor, supporting the conclusion that proteasomes undergo non-selective autophagy-mediated degradation. Similarly, key autophagy proteins such as ATG5, ATG7, and ATG12 were stabilized upon treatment with the proteasome inhibitor MG132, indicating that these proteins are degraded by the UPS. These findings illustrate the mutual regulation between the UPS and autophagy to maintain proteome homeostasis during critical stages of seed development [51].

Several studies indicate that endosperm development is influenced by the HAIKU (IKU) pathway [9,34]. The HAIKU pathway regulates endosperm proliferation and cellularization in Arabidopsis and, therefore, is a key regulator of seed size in Arabidopsis. This pathway consists of three core genes preferentially expressed in early endosperm: *IKU1*, encoding a VQ-motif-containing protein; *MINISEED3* (*MINI3*), encoding a WRKY transcription factor; and *IKU2* (*HAIKU2*), encoding a leucine-rich repeat receptor-like kinase [52,53,54]. Pollination of *iku* mutants with wildtype pollen produced normal-sized seeds, suggesting that *IKU1*, *IKU2*, and *MINI3* regulate seed growth zygotically. Consistently, mutations in these genes resulted in reduced growth and precocious cellularization of the endosperm, resulting in a smaller seed size phenotype [52,53,54]. The transcriptional activator SHB1 (SHORT HYPOCOTYL UNDER BLUE1) works upstream of MINI3 and IKU2 to promote seed enlargement. Indeed, MINI3, together with SHB1, directly binds *MINI3* and *IKU2* promoters to promote their expression [55]. Consistently, dominant *shb1-D* mutant developed larger seeds, and this phenotype is suppressed by *IKU2* or *MIN3* inactivation [56]. Also, IKU1 interacts with MINI3 to positively regulate *MINI3* and *IKU2* directly or in a transcriptional complex together with SHB1 [34,53,56]. Abscisic acid (ABA) regulates seed size by negatively controlling the IKU pathway. Indeed, in the ABA-deficient mutant *aba2*, seed size increased along with the expression of *SHB1*, *MINI3*, and *IKU2*. It has been shown that ABA regulates the IKU pathway by repressing *SHB1* expression via the transcription factor ABI5 (ABA INSENSITIVE 5). Consistently, the *abi5* mutant shows larger seeds and higher *SHB1* transcript levels [57]. Furthermore, it has been reported that BRs also affect seed size through zygotic tissues. Indeed, BR-deficient and BR-insensitive mutants produce small, round seeds, and fertilization of these mutants with wildtype pollen results in normal-sized but round seeds, indicating zygotic control. BR activates the expression of *SHB1*, *IKU1*, *MINI3*, and *IKU2* in Arabidopsis endosperm, and the BR-response transcription factor BRASSINAZOLE-RESISTANT1 (BZR1) binds to the promoters of *SHB1*, *MINI3*, and *IKU2* directly [58].

Downstream, the IKU pathway’s cytokinin signaling acts to control seed growth. In Arabidopsis, inactivation of the cytokinin signaling components, such as the sensor histidine kinases AHKs or the histidine phosphotransferases AHPs, results in an increased seed size [59,60]. Indeed, cytokinin homeostasis can affect seed number and seed size in *Arabidopsis* and crop plants [38,61,62]. Therefore, cytokinin signaling may contribute to balancing seed size and seed number. Interestingly, *CYTOKININ OXIDASE 2* (*CKX2*) is a direct target of MINI3. Indeed, it has been demonstrated that MINI3 binds to the promoter of *CKX2* to regulate its expression, and the seed size phenotype of *iku2* can be partially rescued by the *ahk2 ahk3* mutant or overexpression of *CKX2*. Therefore, the IKU pathway acts, at least partially, through cytokinin signaling to regulate seed size [63].

Recently, it has been shown that ectopic overexpression of *AtSHB1* results in larger seeds in canola, where AtSHB1 regulates the expression of canola genes which are homologous to Arabidopsis *MINI3*, *IKU2*, *SHB1*, *AGAMOUS-LIKE 62* (*AGL62*), and *FERTILIZATION INDEPENDENT ENDOSPERM (FIE*). Consistently, SHB1 regulates *AGL62* and *FIE* in Arabidopsis [64]. These findings suggest that SHB1 may regulate different aspects of seed development, including the activity of the Polycomb Repressive Complex 2 responsible for epigenetic control during seed development (FIS-PRC2) [34,64,65].

Interestingly, the evolutionarily conserved role of IKU2 during endosperm proliferation in dicots and monocots has been recently demonstrated [66]. *IKU2s* was epigenetically repressed by PRC2 in dicot plants, while in monocots, this expression continued during endosperm development due to the lack of H3K27me3 markers on *IKU2s* gene loci [66]. This ancestral *IKU2* function, but divergent epigenetic regulation may reveal the evolutionary route of seed development [66].

### 2.2. Does Cell Wall Constrain Seed Growth?

Many QTL and genes involved in the control of seed size in Arabidopsis and crops have been identified, but how seed growth is arrested once it has reached a definite size is still an open question. In other organisms, the determination of organ size is thought to be controlled by mechanical and biochemical signals [67,68]. Mechanical signals in plants influence growth by the modulation of auxin distribution [69], cytoskeleton organization [70], chromatin organization [71], and gene expression [72]. The primary cause of mechanical stress in plant cells is turgor pressure, defined as the force exerted by water pushing outward on the plasma membrane and the plant cell wall (CW), which counterbalances it. Differences in solute concentration between the outside of the cell membrane and the cytoplasm generate turgor pressure, which puts CW in tension [73]. Plant cells are interconnected by CWs, consequently the build-up of turgor-driven tensile stresses provide instructive intercellular and intertissue signals influencing morphogenesis. Since cells within tissues are not mechanically isolated, mechanical signals encompass both cell-autonomous and noncell-autonomous contributions [74]. For instance, how organ growth is influenced by pressure from inner tissues has been studied in developing seeds. Seed growth depends on the interplay between the testa and the endosperm [7], both maternal-derived tissues with a pivotal role in the control of seed size. Within the endosperm, the rise in turgor pressure, generated by osmolyte accumulation, is believed to drive seed growth, while its progressive reduction at the end of the growth phase was proposed to contribute to growth arrest [75]. Accordingly, nanoindentation techniques have shown that endosperm pressure follows this trend. Surprisingly, *haiku2* mutant fails to decrease endosperm pressure at the end of the growth phase but develops smaller seeds than the WT [76], suggesting that endosperm pressure plays two antagonistic roles: it both drives and inhibits seed growth. This can be explained by the observation that the increase of endosperm pressure exerts tensile stress over the testa, inducing the expression of *EUI-LIKE P450 A1* [77], a negative regulator of seed size, which promotes CW stiffening and thus constrain seed growth [78]. Accordingly stiffening was also observed in wheat grain at late developmental stage which may contribute to arrest grain radial growth [79].

Environmental stresses are well known to reduce cell growth in response to CW stiffening [80]. Accordingly, it has been shown that strain stiffening restricts organ bulging and limits growth in tomato shoots [81]. Moreover, growth arrest in coleoptile has been correlated with loss of CW plasticity rather than turgor pressure, which implies an increase in CW stiffness [82]. This evidence highlights the importance of CW mechanical properties during plant development. The CW is composed of cellulose, the major load-bearing component, non-cellulosic polysaccharides (pectin and hemicellulose), and other non-saccharide components [83]. The pectin matrix is believed to play a central role in the determination of cell-wall mechanical properties. Homogalacturonans (HG) is the most abundant pectin. It is synthesized in a methyl-esterified form, and its degree of methyl esterification is controlled by the action of Pectin Methyl Esterases (PMEs) [84]. The degree of methyl esterification influences HG enzymatic degradation, which leads to cell-wall weakening and promotes growth [85]. On the other hand, full de-methyl esterification causes the formation of cross-links, which increase stiffness and restrain growth [86]. Accordingly, it was observed that during the late seed growth phase, pectin de-methyl esterification occurs mostly in the adaxial epidermis of the outer integument, supporting its load-bearing role [76]. The overexpression of inhibitors of PME activity (PMEI5) results in larger and heavier seeds, a phenotype that could be the consequence of an altered level of pectin methyl esterification or reduced seed production in the silique [87]. Conversely, mutation of PMEI6 produces seeds with reduced width and length, leading to smaller seeds [88], without any variation in the production of seeds in the silique. In grasses, pectin is less abundant than in eudicots [89]. Nevertheless, it plays an important role in seed development. Recently, it was shown in barley that the nucellus, the central part of the ovule, displays a different pattern of HG methyl esterification, more de-esterified in the inner nucellar cells than in the outer one [90]. Mutation of both *OPM1* and *OPM2* (*OVULE PECTIN MODIFIER1* and *2*), belonging to the PME family, increased barley grain length. Consistently, overexpression of *PMEI* had a similar effect, thus strengthening the importance of the degree of HG methyl esterification during seed development [90].

Mutation of genes directly involved in CW biosynthesis rather than CW remodeling seems to affect seed growth and development, altering the final size and weight. For instance, mutation of *CELLULOSE SYNTHASE 2* and *9* (*CESA2* and *CESA9*), which are involved in cellulose biosynthesis, results in smaller seeds [91,92]. In the case of *CESA5*, even though the length and area are reduced, the weight [88] does not seem to be affected [92].

The CW, beside polysaccharides, contains different proteins involved in CW assembly and loosening. Among these, EXPANSINs (EXPNs) are CW-loosening proteins [93]. EXPNs induce CW relaxation by cutting the connection between cellulosic and non-cellulosic polysaccharides, thus promoting cell swelling [94,95]. EXPNs have been shown to promote seed growth in Arabidopsis, maize, rice, and wheat [77,96,97,98,99,100]. Since EXPNs promote wall-loosening, which results in the reduction of turgor pressure and cell swelling, it would be interesting to study how EXPNs affect turgor-driven tensile stresses between tissues, in particular between testa and endosperm.

Plant cells constantly oversee CW integrity (CWI) and, in the case of CW malfunctioning, activate compensatory mechanisms aiming to restore CW functionality and prevent cell death [101]. Cells monitor CWI with different mechanisms. They can perceive (i) plasma membrane stretches through Ca^2+^ and other ion channels, (ii) shrinking or displacement of the plasma membrane through cell-surface sensor kinase, and (iii) cell-wall modification, most likely through receptor kinase belonging to the *Catharanthus roseus* Receptor-Like Kinases (CrRLK1Ls) family [102]. Mutations in sensors involved in CWI perception affect seed growth. For instance, FERONIA (FER), which is involved in cell elongation [103], is an upstream regulator of RAC/ROPs GTPases, membrane-associated proteins acting as molecular transducers of extracellular signals [104,105]. FER represses cell elongation of integument cells, thus negatively controlling seed growth, through the activation of a signaling pathway with Guanine Exchange Factors 1 (GEF1), which stimulates GDP–GTP exchange and activates RAC/ROPs [106]. Notably, FERONIA-like receptors (FLR) in rice also control grain size, with FLR1, FLR2, and FLR8 acting as negative regulators, while FLR15 is a positive one [107]. Also, the receptor-like kinase FEI2, which is involved in response to CW damage downstream of THESEUS1 (THE1) CrRLK1Ls [108], is likely to be involved in the control of grain size, although it plays a positive function, promoting seed growth [88]. Increasing evidence has shown that mutations or chemical treatments impairing CW deposition activate CWI maintenance mechanisms [101], leading to jasmonic acid (JA) production and lignin deposition in a turgor-dependent manner [108,109,110]. JA is bound by the receptor CORONATINE-INSENSITIVE PROTEIN 1 (COI1) [111] and triggers degradation of the JASMONATE-ZIM DOMAIN (JAZ) repressors, thus relieving the JAZ-mediated repression of the transcription factor targets, such as MYCs [112]. JA has been shown to play a pivotal role in the control of seed size. Indeed, both *coi1* and *myc2*/*3*/*4*/*5* mutant seeds show increased size and weight [113], while *JAZ11* overexpression reduces it [114]. Moreover, also the loss-of-function mutant for *JASMONATE RESISTANT 1 (JAR1)*, which catalyzes the formation of bioactive JA-Ile conjugates, shows increased seed size and weight [113]. Conversely, alteration of JA biosynthesis caused by mutation impairing keto-acyl thiolase *2B* (*KAT-2B*) leads to a reduction in seed size and grain weight [115]. Mutation in *KAT-2B* also showed a significant increase in ABA and the induction of genes involved in senescence, suggesting that the alteration in grain size and weight observed in this mutant could be caused by multiple effects.

Since CW properties, mechanical signals, and CWI are tightly interconnected to optimize plant growth and development, it would be interesting to assess whether the suppression of CWI maintenance coupled with CW alteration aimed at reducing stiffening of the testa may be an exploitable strategy to further increase seed weight and quality in crops.

## 3. Seed Size as Determinant of Plant Success

### 3.1. Is Seed Size Related to Tolerance to Environmental Stresses?

Environmental factors such as high/low temperatures, drought stress, light intensity, and soil salinity due to changes in precipitation can have a dramatic impact on seed sizes and composition, thus affecting plant development and reducing crop yields. Nonetheless, the precise molecular mechanisms underlying these variations are still largely unexplored.

Temperature plays a crucial role in plant growth and development, directly influencing seed quality and crop yield. Both extremely high and low temperatures can have detrimental effects on seeds, eventually impacting agricultural productivity. Indeed, temperature fluctuations during seed formation and maturation stages significantly affect seed features and size, often leading to reduced viability and vigor [116]. A recent study highlighted how soybean seeds suffering from high night temperatures during the filling stage are characterized by reduced yield and altered seed size and composition. Three cultivars, ZH39 (high-protein variety), Z1307 (medium-protein variety), and ZH76 (low-protein variety), have been subjected to a 28 °C high night temperature treatment for two years. The treatment had a detrimental effect on seed size in all varieties, as the length, width, and diameter of seeds showed a significant decrease. Along with the seed size, the protein, residue, and oil contents were also affected by the treatment. A transcriptomic analysis carried out on samples of treated soybean leaves revealed that the seed size and quality phenotype following heat treatment was due, as previously suggested, to the down-regulation of both genes involved in regulating pericarp cell-wall expansion [117] and *HEAT SHOCK PROTEINS* (*HSPs*) related genes [116,118,119].

The role of HSPs in relation to heat stress and seed size was further investigated in tobacco, where 61 HSP70 members have been identified. HSP70 is a highly conserved HSP, acting as the main molecular chaperone in most plants [120]. Indeed, *NtHSP70-8* was found to be highly expressed under drought stress conditions, conferring drought tolerance to tobacco when overexpressed [121]. In the study by Zhang et al., the aim was to identify and characterize tobacco HSP70 genes with high homology to *NtHSP70-8*, which could play a crucial role in alleviating heat stress. *NtHSP70-8b* was chosen, and its expression levels under heat, drought, high salt stress, and abscisic acid (ABA) treatment were assessed. ABA is known to play an essential role in heat-stress response [122], as it has been reported that overexpression of the *ABA RESPONSE ELEMENT BINDING* (AREB) gene enhances high-temperature tolerance [123]. The expression of *NtHSP70-8b* reached its highest level after 4 h heat and ABA treatment. Interestingly, *NtHSP70-8b* overexpression significantly increased seed weight and diameter, while knockout lines exhibited the opposite phenotype. In addition, OE lines showed a significant increase in the expression levels of ABA synthesis and response, stress defense, and other HSP genes, resulting in a lower H_2_O_2_ and malondialdehyde (MDA) content, while KO lines showed significantly reduced antioxidant capacity compared to the wildtype under heat-stress conditions [124]. These results suggest that *NtHSP70-8b* has substantial potential for improving tobacco seed yield, as well as tolerance to heat stress.

Among the great number of transcription regulators that plants have evolved to cope with environmental stresses, an interesting example is represented by the TFs belonging to the Alfin-like (AL) family, which are related to seed size and drought stress tolerance in *Oryza sativa* (rice) [125]. Water availability is a more and more significant threat to rice production since the water required for rice cultivation represents approximately 50% of the water used for agricultural purposes [126]. By association analysis, it was shown that the natural variation of *OsALs* genes is mainly associated with seed size and drought tolerance. Thus, two loss-of-function mutants were produced, *osal7.1* and *osal11*. Overexpressing and mutant lines for *OsAL7.1* and *OsAL11* demonstrated that these two proteins negatively regulate both seed size and drought tolerance, although the molecular mechanism of this activity has not yet been unveiled [125].

The *GRAIN SIZE AND ABIOTIC STRESS TOLERANCE 1 (GSA1)* QTL has been identified for its allelic variation on grain size and tolerance to abiotic stresses [127]. As for grain size, natural variation in *GSA1* causes reduced grain weight, width, and length. Based on genetic and molecular analyses, it was proved that GSA1 positively regulates grain size through the control of the ratio between cell proliferation and cell expansion during spikelet development. It has been previously shown that auxin plays a major role in the control of seed size through the activity of a number of auxin regulatory factors, namely BIG GRAIN1 (BG1) and TILLERING AND SMALL GRAIN 1 (TSG1) [128,129]. Consistently, RNA-seq of *GSA1* allelic variants revealed that GSA1 indirectly alters auxin levels and auxin-related genes, as well as the auxin transporter PIN1 [127]. Curiously, *GSA1* encodes for a UDP-glucosyltransferase (UTG83A1), whose enzymatic activity towards flavonoids and monolignols has been proved in vitro [127]. It is known that flavonoid glycosides accumulate under abiotic stresses to protect plants against oxidative damage [130]. A metabolomic assay on the allelic variants of *GSA1* under normal and salt stress conditions revealed that the relative levels of flavonoid glycosides were lower in the more sensitive variants and higher in the more tolerant ones, thus suggesting that GSA1 is crucial for the induction of the phenylpropanoid pathway and the accumulation of flavonoid glycosides and anthocyanins in response to abiotic stress [127].

Photoperiod, as a marker of day length, represents an important environmental cue as it allows plants to undergo developmental and physiological processes during the most favorable period in order to maximize their reproductive success [131]. Plants are classified into long-day (LD), short-day (SD), or day-neutral, depending on their optimal photoperiod for flowering, although photoperiod also influences vegetative development. Recently, it has been shown that growing under the most suitable photoperiodic condition results in bigger and heavier seeds, suggesting that photoperiod-mediated floral transition and seed development may be synergistically controlled by conserved regulatory mechanisms [132].

Given the central role of the regulator CONSTANS (CO) in integrating internal and external signals into the photoperiodic flowering pathway, it was investigated its contribution to the post-flowering reproductive growth in Arabidopsis [132]. Using different transgenic lines, it was possible to observe that overexpressing and mutant lines for CO resulted in larger and smaller seed phenotypes, respectively, under both SD and LD and that the mutant line *co-9* was insensitive to day length.

Moreover, using a steroid-inducible version of *CO*, activation at 6 Days After Pollination (DAP) resulted in significantly larger seeds, while at 8 DAP did not affect seed size, suggesting that CO has a key role in the photo-mediated early stage of seed development, with its expression pattern pointing to an action during early seed coat and/or embryo development. Transcriptomic analysis revealed that CO represses *APETALA 2* (*AP2*) transcription, thus restraining its negative effect on seed size during the early stages of seed development [132]. Therefore, CO promotes seed enlargement in photoperiodic plants under optimal photoperiod conditions, exerting its function by recruiting different co-factors to inversely regulate *AP2* expression in a day length-dependent manner. While this model was proved in Arabidopsis and rice, soybean CO homolog only has a light effect on seed size as on the expression of *AP2*, implying that the mechanism identified may not be evolutionarily conserved [132] (Figure 1).

### 3.2. Seed Size Is a Quantitative and Qualitative Agronomic Trait for Cereals

**Figure 2:**
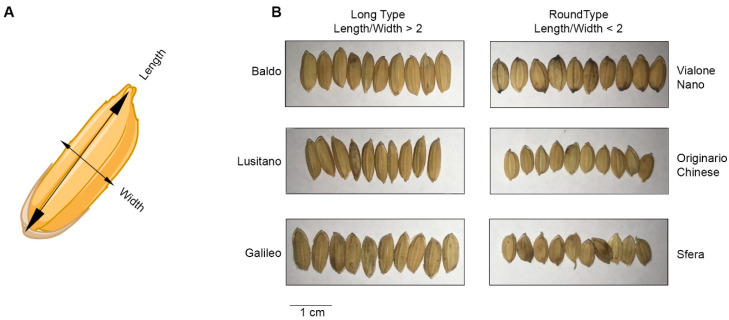
Length/Width ratio of seed is an agronomic trait under natural and artificial selection. (**A**) The two dimensions that determine the seed size. The length is the maximum of the longest axis of the seed, while the width is measured perpendicular to the length axis. (**B**) 10-grain of some Italian rice varieties belonging to the japonica subspecies showing different length/width ratios. Long-type grains are usually characterized by a length/width ratio bigger than 2, while round types are by a ratio lower than 2.

The study of how seed size is established is a fascinating question of plant developmental biology; nevertheless, seed size also represents a relevant qualitative and quantitative agronomic trait that has been the target of natural selection and intensive breeding activity. The value of this trait is due to its contribution to seed weight, one of the criteria that determines final yield in many crops, especially for cereals, which are the main source of calories for humans and animals worldwide. On the other hand, for crops such as rice, seed size represents a valuable qualitative trait. In fact, grain shape and size are important characteristics driving consumers’ choices; hence, they determine the success and price of a rice variety on the market. The two main rice subspecies are japonica and indica, which show different seed shapes: indica grains are characterized by a long and thin shape, while japonica seeds are roundish and short, as the result of many years of domestication and natural selection. However, breeders keep working on always finding new varieties with different length/width ratios and, given that Italy is the first producer of rice in Europe, a beautiful example of breeders’ work can be seen in the grain diversity of the Italian rice varieties belonging to the japonica subspecies (Figure 2) [133].

Breeding activity has benefited from Genome-Wide Association Studies (GWAS) and QTL analyses that helped to understand the natural and artificial selection that contributed to seed size, as well as to decipher the molecular mechanisms, providing targetable genes for new breeding. GWAS is a high throughput analysis that allows the matching of specific genomic variations with the agronomic trait of interest. Recently, Chen and co-authors used GWAS to identify the genetic variants that determine the differences between japonica and indica rice [134]. They found that the coding sequence of the *GRAIN SHAPE ON CHROMOSOME 9 (GSE9)* gene originated de novo in the japonica subspecies from an intergenic region. In fact, the majority of the indica subspecies have an SNP changing A to G at the start code of *GSE9*, implying that they do not express this gene, while japonica subspecies do. Functional analyses confirmed that the japonica *GSE9* gene makes larger seeds because it affects the number and size of the cells of the spikelet hull, regulating cell cycle and expansion. However, the molecular function of GSE9 is still unknown, and hence, further analyses are necessary to discover the molecular mechanism [134].

The most diffuse strategy for the identification of the loci linked to an agronomic trait is still the QTL analysis. It relies on the mapping of genetic markers to identify the genomic locus segregating with a specific quantitative trait. Over the years, many QTLs have been identified for grain size, but only a few of them have been characterized. Hence, here we describe the main QTLs for which extensive functional analyses have been done, while a more extensive list of the QTLs associated with grain size is reported in Table 1 [127,135,136,137,138,139].

The first QTL associated with grain size in the indica subspecies was identified on the centromeric region of chromosome 3, and alone, it explained 20% of the weight variations and 55% of the length variations in grain rice varieties. In 2006, this QTL was identified as *GRAIN SIZE 3* (*GS3*) locus [140], which encodes for a Gγ subunit (Group III) of the heterotrimeric G-proteins, a protein family extensively shown to be regulators of grain size. The two main domains of the GS3 protein are the Gy-like domain at the N-terminal part and the Cys-domain at the C-terminal [147]. The Gy-like domain was already known as the Organ Size Regulator (OSR) because it was recognized to be specific and sufficient for the negative function of GS3 in grain size [15]. In fact, the Gy-like domain participates in the binding of GS3 with Gβ-protein RGB, which, together with other G-proteins, such as DENSE AND ERECT PANICLE 1 (DEP1) and RICE Gα SUBUNIT (RGA), regulate seed size.

A mutation widely present in rice varieties with long grain changes the C to A, inserting a premature stop coding and producing a truncated protein missing most of the Gγ-like domain2 and the Cys-domain [15]. This indicates that the mutation has ancient origins and that GS3 works as a negative regulator of seed size, as also confirmed by several functional analyses. Another interesting mutation of GS3 was found in the Chuan-7 rice variety that lacks only the Cys-domain and determines extra small seeds, suggesting that the Cys-domain is a regulatory region for GS3 function [15,148]. In 2021, Yang and co-authors found that the Cys-domain is essential for the interaction with the RING E3 ligase Chang Li Geng1-1 (CLG1), which brings GS3 to degradation [149]. Moreover, genetic analyses revealed that CLG1 natural variations are associated with different seed lengths, further supporting the importance of the GS3-mediated pathway in the regulation of grain size.

Another well-studied QTL is called *GRAIN WIDTH* 5 (*GW5*, also known as *qSW5* and *GSE5*) [150,151]. This QTL is mainly associated with grain width, which seems to depend on the expression levels of the *GW5* gene [151,152]: high *GW5* expression determines thin grains, while low expression roundish ones. GW5 is used as a marker for distinguishing japonica and indica subspecies, as deletions in the promoter of *GW5* are typical of the japonica seeds with a roundish phenotype [141,151,152]. At the molecular level, *GW5* encodes for a calmodulin-containing protein localized at the plasma membrane and in the nucleus [142,146], and many pieces of evidence suggest that GW5 controls seed shape, regulating the BR pathway. As well as overall plant growth, BR is also involved in the control of seed size, regulating cell elongation, division, and differentiation [153]. GW5 mediates BR signaling because it interacts with the GLYCOGEN SYNTHASE KINASES (GSK2, the homologous of the Arabidopsis BIN2), inhibiting its activity, which includes the phosphorylation of two transcription factors, namely BZR1 and DWARF AND LOW-TILLERING (DLT) [142]. The GSK2-mediated phosphorylation of BZR1 and DLT determines their degradation and hence restrains BR-mediated gene expression.

Moreover, GW5 interacts with the transcription factor WRKY53 [154] to control the expression of the *SGW5* gene, a novel QTL linked with grain width [146]. Given that WRKY53 is a transcription factor involved in BR signaling to regulate seed size in rice [154], this could suggest that GW5 participates in the BR-mediated gene expression regulating different factors. However, how the GW5-mediated BR pathway determines the length/width ratio of the seed is still largely unknown. In particular, it would be interesting to understand the effect of GW5-mediated BR pathway at single-cell resolution, also considering the impact of BR on seed size as described above, and the identification of other QTLs, such as GS10 and GL2, both linking seed size with the BR pathway [144,145,155].

A deep functional characterization has been carried out for the GRAIN WIDTH AND WEIGHT 2 (GW2) QTL, which encodes for an E3 ligase protein and is homologous to the already mentioned Arabidopsis DA2 protein [13]. GW2 was found in a QTLs mapping from the cross between the japonica variety WY3, with large grain, and the indica one FAZ1, with small seeds. The analysis revealed that the GW2 allele of the WY3 rice is truncated, while the one from FAZ1 rice encodes the full-length protein that negatively regulates seed width [143]. The size-related phenotype is likely to be due to the cell number of the spikelet hulls, suggesting that GW2 inhibits cell division, an observation also confirmed by a new GW2 allele called *gw2.1* [156]. However, the molecular mechanism underlying GW2-mediated cell proliferation is not fully elucidated yet, although, given the nature of the protein, finding its target of degradation could be the goal. In fact, E3 ligase activity provides specificity for proteasome degradation, recognizing and ubiquitinating only specific targets, but finding the E3 ligase-target match can be very challenging due to the transient nature of the binding. So far, only two targets have been identified for GW2: EXPLA1, an expansive, driving cell-wall loosening [157], and WG1, a CC-type glutaredoxin protein that positively regulated cell proliferation [158].

Moreover, the size-related phenotype, GW2, has also been characterized for the effect on endosperm expansion, which is an indirect effect of the spikelet hull size. Endosperm is a critical tissue for grain quality because it accumulates starch, proteins, and lipids as reserve molecules in a process called grain filling [159]. Given the importance of rice as a nutritional food, it is easy to imagine why finding QTL involved in grain filling is an attractive objective, and GW2 has become the focus of many research lines. In 2021, it was shown that the knockout of GW2 by CRISPR-Cas9 positively affects grain protein and minerals contents [160], further supporting the idea that GW2 represents a key locus for enhancing rice quality.

As already mentioned, seeds represent the key element for plant fitness, and many aspects of seed biology need to be considered when agronomic selections are applied. Understanding how seed morphology is linked, for example, to seed vigor, meant as seed longevity and germination, is a fascinating issue that will be discussed in the next paragraph.

### 3.3. Does Seed Size Influence the Success of Seed Germination?

The germination process starts with water uptake and ends with the protrusion of the radicle. Successful seed germination is important for initiating the next phase of the plant life cycle, ensuring vigorous and healthy seedling growth, and ultimately resulting in high crop yields [161]. Additionally, seed size and weight are important agronomic traits influencing plant fitness, environmental stress adaptation, yield, and quality [162,163,164]. In barley, ABA inhibits GA-mediated α-amylase expression to control dormancy and germination [165]. Similarly, the study by Asatsuma et al. [166] found that rice lines with suppressed expression of α-amylase I-1 at both the mRNA and protein levels exhibited significantly delayed seed germination and seedling growth [166]. Seed development is a complex process, finely regulated by numerous genes that respond to developmental and environmental signals, including ABA, which regulates different processes in plants, such as seed germination, dormancy, fruit development, and stress adaptation [9,167,168]. Variations in ABA levels are mainly controlled by de novo biosynthesis, hydroxylation, and conjugation. Notably, the hydrolysis of ABA-glucose ester mediated by β-glucosidase, which releases active ABA, is considered faster than the de novo biosynthesis pathway and plays a key role in regulating endogenous ABA content in response to abiotic stresses, thus influencing seed physiology [169].

In a recent study by Wang et al., the *Citrullus lanatus* β-glucosidases 1 (*ClBG1*) gene, which encodes a β-glucosidase involved in ABA modulation, was found to be highly expressed during the ripening of cultivated watermelon but at low levels in non-ripening wild watermelon, suggesting its involvement in ABA-mediated fruit ripening. To further investigate the function of *ClBG1* in watermelon, the CRISPR/Cas9 system was used to introduce mutations in the *ClBG1* gene in the cultivated watermelon variety ZXJM. This study found that dysfunction of *ClBG1* led to decreased seed size and weight. Notably, *ClBG1*, located on chromosome 8, is physically near the seed size QTL ClSS8.2 [170,171]. This finding confirms that *ClBG1* may play a role in watermelon seed size control. Additionally, the shape and size of plant organs are controlled by programmed cell division and cell expansion. Specifically, the spatiotemporal patterns of cell division and cell enlargement are mainly regulated by the reorientation of microtubule arrays. In this study, the GO term enrichment analysis of the DEGs revealed that microtubule-, tubulin-, and cell cycle-related genes were strongly affected by mutation in the *Clbg1* gene, which was consistent with the alteration of cell number and size. Although cell size was significantly increased, seed size and weight decreased in the *Clbg1*-mutant lines, indicating that cell number is the most important factor in seed size determination [172]. In recent years, there has been substantial progress in identifying important genes that regulate grain number and weight, significantly advancing the genetic improvement of rice yield [173]. To date, over 80 genes related to rice grain size have been identified and linked to various regulatory pathways. As already mentioned, a significant pathway is the G-protein signaling pathway, which includes the Heterotrimeric G-protein α subunit (*RGA1*) [15,174]. The *RGA1* gene plays a crucial role in controlling several seed traits in rice, including grain size and seed germination, primarily through modulating ABA content [175]. The mutation in *SMALL AND ROUND GRAIN 5* (*SRG5*) gene in rice is associated with increased ABA levels, which correlate with delayed seed germination. RT-qPCR analysis indicated a significant decrease in ABA biosynthesis and catabolism gene expression in the *srg5* mutant, with biosynthetic genes at about 70% and catabolic genes at 40% of wildtype levels. This was further demonstrated by a 20% increase in ABA levels in the *srg5* mutant in germinating seeds compared to wildtype. To identify the target gene of *SRG5*, BSA, and genetic linkage analysis pinpointed the Alpha-subunit of GTP-binding protein Os05g0333200, which encodes *RGA1*, as the gene responsible for the small-grain phenotype in the *srg5* mutant. A G to A mutation in the first intron of *RGA1* disrupted normal splicing, leading to a premature stop codon and a truncated protein [175]. This mutation in *RGA1* resulted in small grain size and reduced plant height, confirming it as the target gene affected by the *srg5* mutation. Thus, the effect of RGA1 extends beyond seed size to include significant impacts on seed germination, mediated through regulating ABA catabolism [175].

Seed germination and post-germination seedling performance are often affected by environmental factors and inherent seed traits. Likewise, seed size (mass) is an important evolutionary trait that affects seed germination and seedling performance [176,177,178,179]. In phenotypic analysis concerning seed size and germination, it is evident that seed size plays a key role in germination outcomes under varied environmental conditions. Larger seeds have greater resources for seedling metabolism to utilize during germination and an increased ability to stress tolerance, enabling greater seedling survival and growth [180]. In *Brassica tournefortii* and *Lotus garcinii*, larger seeds exhibited a notably higher germination percentage compared to smaller seeds under controlled temperature. Specifically, larger seeds demonstrated enhanced germination under a 12-h light cycle at 25/35 °C and 15/25 °C, respectively. The low germination percentage for small seeds of *B. tournefortii* and *L. garcinii* compared with the large seeds may be attributed to different levels of seed dormancy within a seed lot, as all of the ungerminated seeds were healthy and hence considered viable, which indicates that smaller seeds have a deeper degree of dormancy than larger seeds [181]. Another study on *Dennettia tripetala* investigated the effects of seed size on germination and early seedling growth by categorizing seeds into small, medium, and large sizes. The experiment used a Completely Randomized Design and assessed variables such as seedling height, collar diameter, leaf number, and biomass. The results demonstrated that larger seeds exhibited a slightly delayed germination but improved overall growth characteristics compared to smaller seeds. Indeed, larger seeds showed advantages in seedling height, collar diameter, and biomass, suggesting a larger number of nutrient reserves promoting early growth. Conversely, smaller seeds germinated slightly earlier but displayed lower growth features. These findings contribute to a better understanding of the physiological impacts of seed size on plant development, providing valuable insights for agricultural practices, especially in optimizing the cultivation and regeneration of this species [182].

In natural variation studies, it is evident that larger seeds generally yield better germination and early growth. However, these studies primarily document phenotypic observations and do not explore the underlying genetic pathways that lead to these differences [181,182,183]. This is in contrast to the detailed genetic insights provided by CRISPR-based research, such as the studies on watermelon and rice, where specific genes like *ClBG1* and *RGA1* were targeted, demonstrating how genetic modifications can directly impact seed size and germination processes [172,175]. It is important to understand the complex genetic networks that influence seed size and yield, highlighting how both genetic mechanisms and environmental factors shape these traits [184].

To effectively bridge the gap between the observed phenotypic variations in naturally occurring seed sizes and the precise genetic variations achieved through CRISPR, future research should focus on applying genomic and molecular biology tools to explore the genetic foundations of naturally varying seed sizes.

## Figures and Tables

**Table 1:** List of the main QTLs associated with grain size in rice. The locus is referred to as the RAP-DB ID. The molecular function for the gene causative of the QTL is reported, as well as the effect on length and width, which are the two main dimensions affecting grain size. The main references reporting the QTLs characterization are cited.

QTL	Locus	Molecular Function	Effect on Seed Size	References
**GS3**	Os03g0407400	G-protein	Negative on length	[15,140]
**GW5**	Os05g0187500	Regulator of the BR signaling pathway	Negative on width	[141,142]
**GW2**	Os02g0244100	E3 Ubiquitin Ligase	Negative on width	[143]
**GL6**	Os06g0666100	Transcription factor	Positive on length	[135]
**GL7**	Os07g0603300	Regulator of cell cycle (to be confirmed)	Positive on length	[136]
**GL3.1**	Os03g0646900	Serine/Threonine phosphatase	Positive on length	[137]
**GS3.1**	Os03g0229500	MATE transporter	Negative on length and width	[138]
**GW6**	Os06g0266800	GAST family protein	Positive on width	[139]
**GL2**	Os02g0701300	Transcription factor	Positive on length	[144]
**GS10**	Os10g0522601	Proteins with Armadillo tandem repeats	Negative on width	[145]
**SGW5**	Os02g0543400	WD40 repeat-like domain-containing protein	Positive on width	[146]
**GSA1**	Os03g0757500	UDP-glucosyltransferase	Positive on width and length	[127]
